# Sunscreen-Assisted Selective Photochemical Transformations

**DOI:** 10.3390/molecules25092125

**Published:** 2020-05-01

**Authors:** Or Eivgi, N. Gabriel Lemcoff

**Affiliations:** 1 Department of Chemistry, Ben-Gurion University of the Negev, Beer-Sheva 84105, Israel; 2 Ilse Katz Institute for Nanoscale Science and Technology, Ben-Gurion University of the Negev, Beer-Sheva 84105, Israel

**Keywords:** photochemistry, sunscreen, chromatic selectivity, photolabile protecting groups, photoinduced olefin metathesis

## Abstract

In this review, we describe a simple and general procedure to accomplish selective photochemical reaction sequences for two chromophores that are responsive to similar light frequencies. The essence of the method is based on the exploitation of differences in the molar absorptivity at certain wavelengths of the photosensitive groups, which is enhanced by utilizing light-absorbing auxiliary filter molecules, or “sunscreens”. Thus, the filter molecule hinders the reaction pathway of the least absorbing molecule or group, allowing for the selective reaction of the other. The method was applied to various photochemical reactions, from photolabile protecting group removal to catalytic photoinduced olefin metathesis in different wavelengths and using different sunscreen molecules. Additionally, the sunscreens were shown to be effective also when applied externally to the reaction mixture, avoiding any potential chemical interactions between sunscreen and substrates and circumventing the need to remove the light-filtering molecules from the reaction mixture, adding to the simplicity and generality of the method.

## 1. Introduction

The use of light to carry out organic transformations has resurged dramatically in recent years [1]. Naturally, light is considered a ‘green’ and clean source of energy that can be easily applied and manipulated. In this decade, we witnessed the rebirth of “classic” photochemical reactions [2,3,4] along with the development of novel organic and organometallic photocatalysts for a variety of transformations [5,6,7,8,9,10]. Light is also used to make new materials, for example, in additive manufacturing and stereolithographic processes—where light is applied with excellent spatial resolution and precision [11,12]. Additionally, the development of continuous flow techniques for photochemistry has made the execution of large-scale light-induced processes possible, overcoming the light transmittance issues associated with large-scale photochemical reactions [13,14,15,16,17]. However, when more than one photosensitive group is present in a reaction mixture, the selective triggering of each chromophore may be difficult to accomplish, especially in cases where the absorption spectra of the photosensitive moieties overlap [18]. The photosensitivity of a chromophore at a certain wavelength strongly depends on two factors: its molar absorption coefficient and the quantum yield of the photochemical reaction. In recent years, several chromatic orthogonal systems where two photosensitive moieties may be reacted in a commutative fashion with different colors of light have been designed [19]. Notable works in this field were reported by Bochet et al. [20,21], for example by harnessing kinetic isotope effects to achieve chromatic orthogonality between two types of 2-nitrobenzyl based photolabile protecting groups (PPGs) [22] and even demonstrating the chromatic orthogonal total synthesis of a natural product [23]. Additional significant advancements in the field were reported by Barner-Kowollik et al., who developed a series of chromatic orthogonal photocyclization reactions with applications in polymer chemistry and material science [24,25,26,27,28]. In addition, several chromatic selective systems where two chromophores may be reacted sequentially have been developed with great efficiency [29,30,31,32]. In this focus review, we describe a different approach that was developed in our laboratory to achieve selectivity between two photosensitive groups or photochemical reactions that occur at similar wavelengths. The method exploits differences in the molar absorption coefficient between two chromophores with the aid of an external molecule that acts as a light filter, or as we call it, a “sunscreen”. The filter molecule deters one of the photochemical reactions, prompting a selective sequence. The generality of this method was studied with different types of reactions ranging from PPG removal to light-induced catalytic olefin metathesis reactions and demonstrated with several wavelengths and different filter molecules.

## 2. Development of the Method

**Scheme 1:**
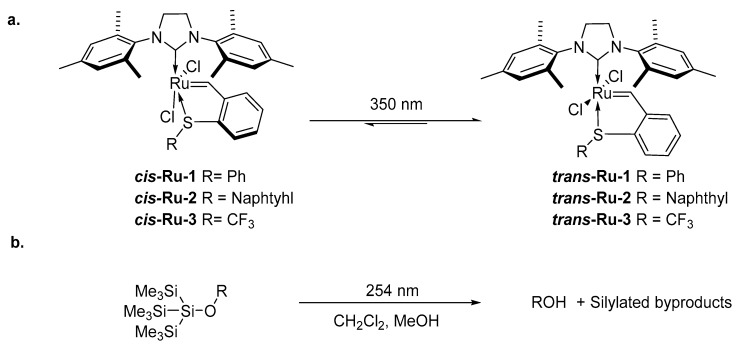
(**a**) Photoisomerization reaction of sulfur chelated ruthenium benzylidene metathesis catalyst **cis-Ru-1** and ***cis-*Ru-2** with UV-A light. (**b**) Photocleavage of supersilyl ethers with UV-C light.

The motivation to develop the method originated in our efforts to design a regioselective chromatic orthogonal ring closing metathesis scheme using photoswitchable olefin metathesis catalysts [33]. The irradiation of metathesis catalysts ***cis-*Ru-1**, ***cis*-Ru-2** or ***cis-*Ru-3** with UV-A light triggers the isomerization of the catalysts from their latent *cis*-dichloro configuration to the metathesis active *trans*-dichloro configuration (Scheme 1a) [34]. This reaction can be coupled with the photocleavage of the tris(trimethylsilyl)silyl ether (“supersilyl ether”) that occurs with UV-C light (Scheme 1b) [35,36].

**Scheme 2:**
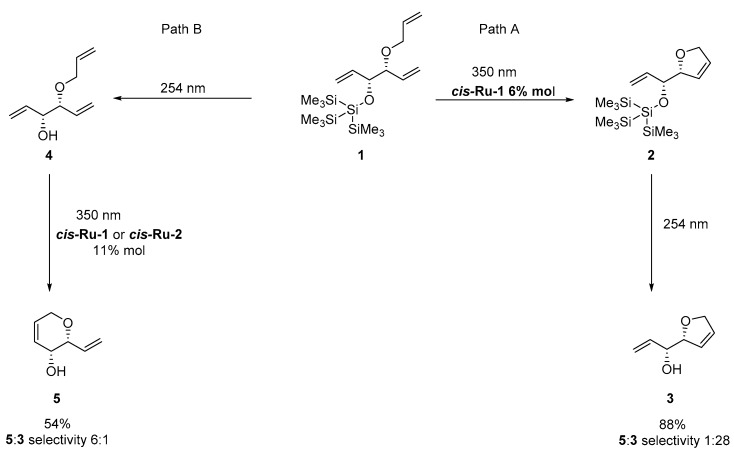
Regioselective chromatic orthogonal sequence for the synthesis of dihydrofuran and dihydropyran.

Inspired by the work of Schmidt et al. [37,38], we envisioned that triene **1**, equipped with the supersilyl group, should undergo ring-closing metathesis (RCM) reactions to form a five-membered ring product **3** (path A) and a six-membered ring product **5** (path B), based on the irradiation sequence (Scheme 2).

When the RCM reaction occurs prior to the cleavage of the supersilyl ether, the steric stress exerted by the bulky PPG favors the formation of a five-membered ring dihydrofuran (path A). Alternatively, the irradiation sequence can be reversed, triggering the removal of the PPG prior to the olefin metathesis reaction. In this scenario, the RCM reaction results in the formation of a six-membered ring product (path B). This scheme provided excellent results in an open system (where the reagents are added before each step); however, the “closed system” scheme (where all the necessary reagents are added from the start) was found to be much more challenging and required significant optimization. The limiting step in the “closed system” was the removal of the photolabile supersilyl ether in the presence of the ruthenium catalyst; this was because although the ruthenium catalyst is not reactive with UV-C light, it still has a high absorption in this region of the UV spectrum (ε^254^: 10^5^ M^−1^·cm^−1^), while the supersilyl protected triene absorbs significantly less (ε^254^: 10^4^ M^−1^·cm^−1^). Thus, it was our observation that the differences in the molar absorption coefficients significantly hampered the photocleavage of the supersilyl ether.

**Figure 1:**
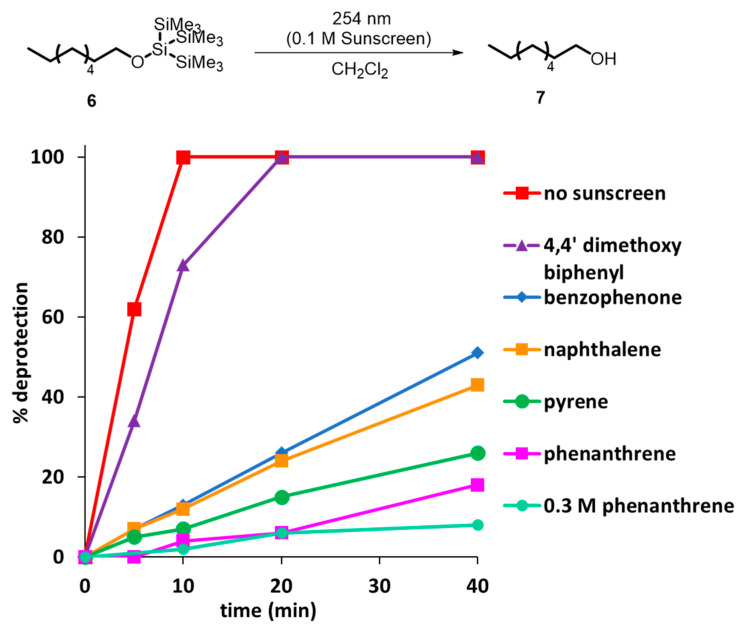
Effect of different additives (0.1 M) on the photocleavage rate of supersilyl protected octanol in 254 nm.

The premise that the ruthenium catalyst “protected” the photosensitive group from reacting by absorbing incident light initiated a study in our lab to explore the nature of this “sunscreen effect”. Since the supersilyl ether is a PPG for alcohols, we sought to exploit this effect to bring about the selective removal of PPGs [39]. Moreover, other light-absorbing molecules were examined to test their “sunscreen effect” efficiency on the UV-C photocleavage of supersilyl ethers (Figure 1).

**Figure 2:**
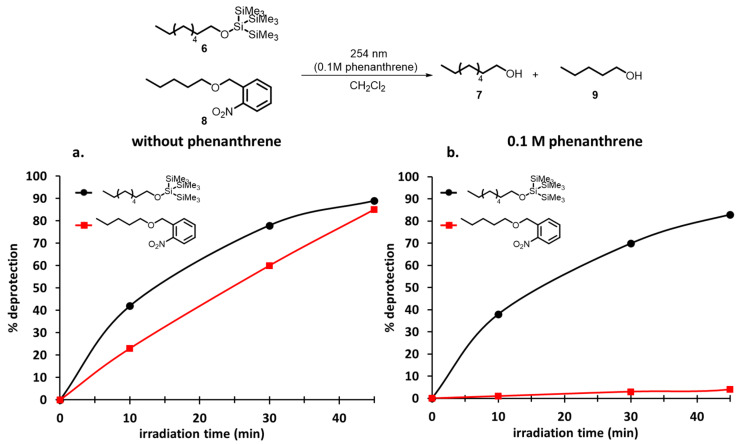
Photodeprotection reaction of 2-nitrobenzyl protected alcohols without phenanthrene (**a**) and with 0.1M phenanthrene (**b**). Reaction conversions measured using ^1^H-NMR.

This set of experiments disclosed that phenanthrene had the most significant effect on slowing the rate of photodeprotection of the supersilyl ethers. Once a suitable filter molecule was chosen, a complementary PPG that could be readily photolyzed in the presence of phenanthrene was sought. The 2-nitrobenzyl group is frequently used as a protecting group in organic chemistry for many functional groups [40]. Using this group, alcohols may be masked as ethers or carbonates, acids as esters, and amines as carbamates [41,42,43,44]. The scaffold of the 2-nitrobenzyl PPG is versatile and by simple modification on the benzylic position or the aromatic ring, the quantum yield of the photochemical reaction and the wavelength of activation can be changed. The molar absorption coefficient of simple unsubstituted 2-nitrobenzyl ethers is about 5 × 10^3^ M^−1^·cm^−1^, which is between 5 and 10-fold larger in comparison to the supersilyl counterpart. Thus, an experiment was set up studying the selective photodeprotection of 2-nitrobenzyl and supersilyl PPGs in the presence of phenanthrene. Satisfyingly, a dichloromethane solution of protected pentanol and octanol with 0. 1 M phenanthrene resulted in a selective removal of the 2-nitrobenzyl PPG, while the supersilyl group remained intact when exposed to 254 nm UV light (Figure 2).

**Figure 3:**
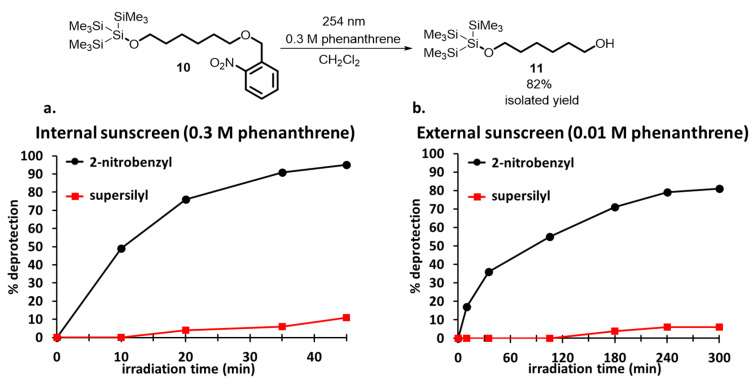
(**a**) Selective removal of 2-nitrobenzyl from a masked 1,6 hexanediol linker, using 0.3 M phenanthrene (82% isolated yield). (**b**) Selective removal of 2-nitrobenzyl using an external solution of 0.01 M phenanthrene.

To further investigate the role of the phenanthrene, we examined how an external solution of phenanthrene would affect the reaction selectivity. The “external sunscreen” scheme was also successful, and selective removal of the more absorbing PPG was clearly observed. The use of an external sunscreen provides several advantages, such as no need to purify the product from the sunscreen, easy tunability of the absorption properties by just changing the concentration of the external solution, and it being very simple to reuse the same sunscreen after the reaction is over (no waste). Moreover, the selective removal of 2-nitrobenzyl was achieved also when both chromophores were on the same molecular scaffold. This was demonstrated with bis-phenol-A and 1,6 hexane diol-based linkers and with the sunscreen applied either internally or externally (Figure 3).

**Scheme 3:**
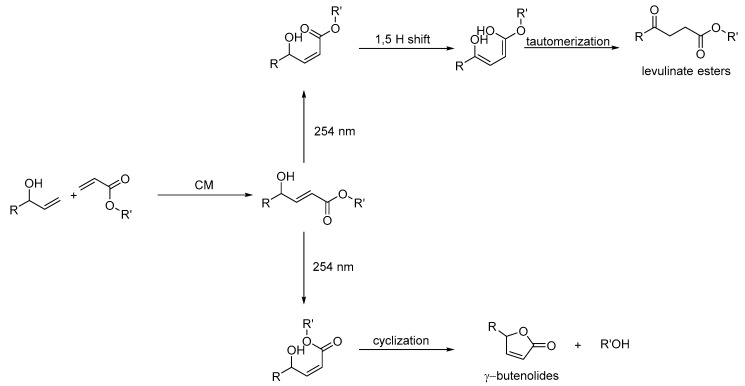
Formation of γ-butenolides and levulinate esters from the CM product of allyl alcohols and acrylate esters in a photochemically divergent reaction.

The successful suppression of the photocleavage of supersilyl ethers inspired us to apply this strategy to affect other photochemical reactions [45]. The cross-metathesis (CM) product of allylic and acrylic substrates may undergo two competing photochemical reactions when exposed to 254 nm light to form γ-butenolides or levulinate esters (Scheme 3). The reaction to form the butenolide scaffold involves a *trans* to *cis* double-bond isomerization and concomitant intramolecular lactonization. The formation of the levulinate esters begins also with a *trans* to *cis* double-bond isomerization accompanied by a photochemical 1,5 hydrogen shift and subsequent tautomerization.

**Figure 4:**
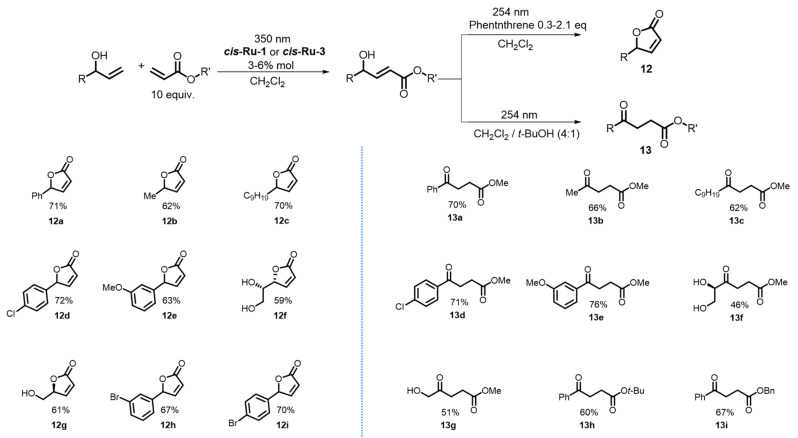
Photochemical divergent synthesis of butenolides and levulinate esters: isolated yields.

**Scheme 4:**
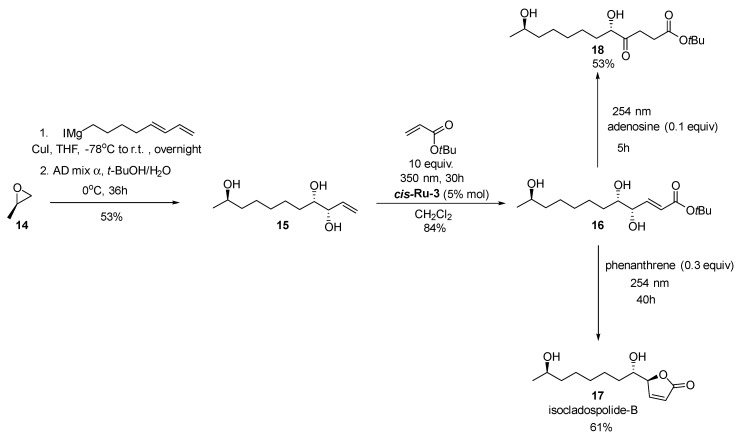
Synthesis of isocladospolide B and its corresponding levulinate ester by photochemical divergent reaction modulated by phenanthrene.

These two photochemical processes are not selective, yielding approximately equimolar amounts of both butenolide and levulinate products [46]. Interestingly, when phenanthrene was added to the reaction mixture in dichloromethane, illumination with 254 nm resulted in the formation of butenolides with high selectivity and yields. Without phenanthrene and with the addition of *t*-butanol as a co-solvent (1:4 *t-*butanol:CH_2_Cl_2_), the selectivity was reversed toward the formation of the levulinate ester. To verify that indeed that the role of phenanthrene is to block the 1,5-hydrogen shift, Stern-Volmer experiments were conducted to rule out sensitization or quenching effects caused by the phenanthrene. Thus, a complete selective photochemical protocol for the synthesis of butenolides and levulinate esters was developed (Scheme 4). The first step was a photochemical CM reaction with 350 nm light using the photoswitchable catalysts ***cis-*Ru-1** or ***cis-*****Ru-3**. Then, the metathesis product was exposed to 254 nm light in the presence or the absence of phenanthrene to furnish the desired product. Using this protocol, a large number of butenolides and levulinates were prepared (Figure 4) in high yields and selectivity.

Moreover, using this photochemical protocol, a total synthesis of a marine natural product, *iso*-cladospolide B [47], was completed in fewer steps and higher overall yields compared to other syntheses in the literature (Scheme 4) [48].

**Figure 5:**
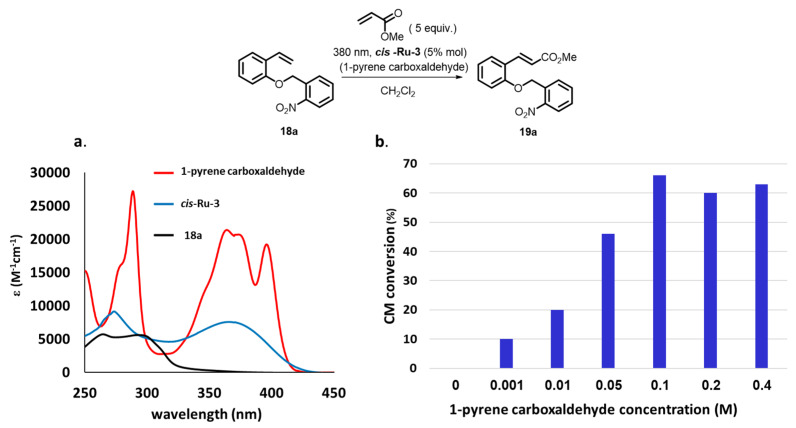
(**a**) UV spectra of **18a**, ***cis*-Ru-3** and 1-pyrenecarboxaldehyde (**b**) CM conversions of **18a** and methyl acrylate with different 1-pyrene carboxaldehyde concentrations.

The possibility to use phenanthrene to modulate the selectivities of photochemical reactions with UV-C light encouraged us to extend this concept to different wavelengths. 2-Hydroxy styrenes are considered impractical for olefin metathesis as they form a stable chelate with the ruthenium metal center [49,50]. In order to use this type of olefins in metathesis reactions, the phenol functionality needs to be masked [51]. To address this problem, we planned to use the sunscreen methodology and protect the phenol functionality with a PPG. By using a suitable filter molecule, we aimed to selectively activate a photoswitchable metathesis catalyst, exploiting the strong absorption of the ruthenium catalysts in the UV-A region [52]. As a sunscreen, we selected 1-pyrenecarboxaldehyde, which is a dye known for its strong absorption in the UV-A region, and in order to avoid undesired side reactions, the 1-pyrene carboxaldehyde filter was only used externally. The PPG selected to mask the phenol functionality was 2-nitrobenzyl, given its relatively low absorption in the UV-A region (Figure 5a). The applicability of this scheme was tested on the CM reaction of 2-nitrobenzyl protected 2-hydroxy styrene and methyl acrylate (Figure 5b).

**Figure 6:**
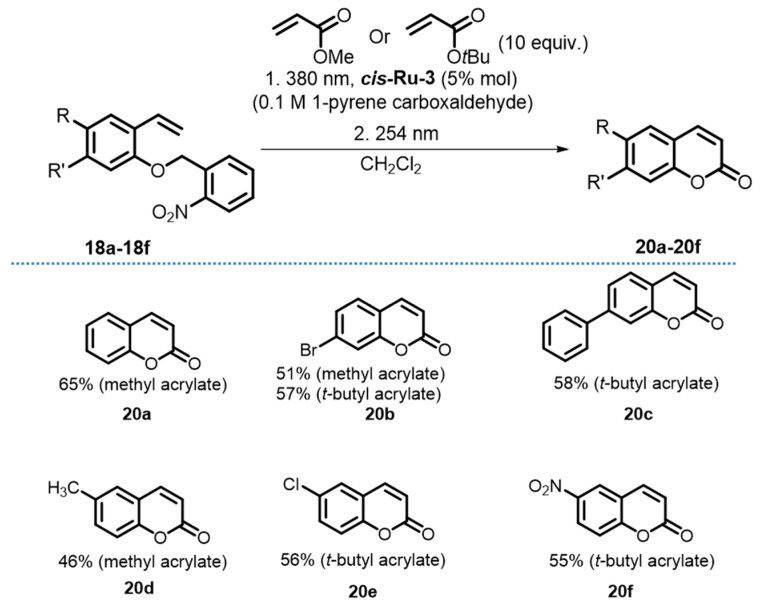
Photochemical synthesis of coumarin derivatives via sunscreen-enabled olefin metathesis: isolated yields.

As expected, without the external sunscreen solution, no conversion was observed as the catalyst rapidly decomposed. It is important to highlight that only very low concentrations of catalyst are activated at a given time; this means that small amounts of free phenol will be enough to decompose all of the active catalyst and stop the reaction. The conversions were improved by increasing the 1-pyrene carboxaldehyde solution concentration until maximum conversion was observed with 0.1 M of 1-pyrenecarboxaldehyde, proving the efficacy of the sunscreen method even for catalytic reactions. This sunscreen-enabled photoinduced olefin metathesis was applied in a bichromatic synthesis of coumarins (Figure 6). The first step of the synthesis was the CM reaction between various 2-nitrobenzyl protected styrenes and acrylates at 380 nm in the presence of 1-pyrene carboxaldehyde external solution. After the metathesis reaction was completed, the external filter solution was removed, and the reaction mixture was exposed to 254 nm, triggering a chain of three photochemical reactions. First, photocleavage of the nitrobenzyl PPG, then, a *trans* to *cis* double bond isomerization, and finally cyclization to furnish the coumarin compounds.

**Figure 7:**
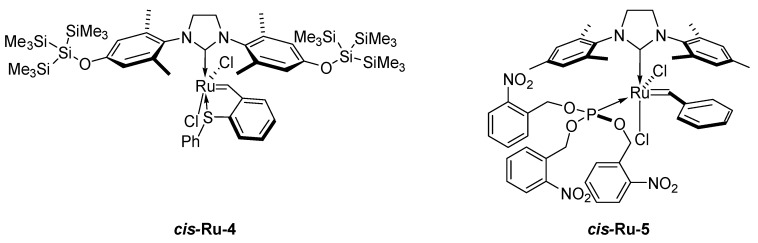
Photoswitchable metathesis catalysts with chromatic orthogonal self-destruction switches.

The possibility to selectively activate a metathesis catalyst in the presence of PPGs inspired us to look for new avenues in photoinduced latent catalysts design [53,54,55]. For this reason, we recently developed and prepared a metathesis catalyst bearing a chromatic orthogonal kill switch, based on the natural chromatic orthogonality of the photoisomerization reaction of the sulfur chelated ruthenium benzylidene metathesis catalysts and the cleavage of supersilyl ethers [46]. Thus, a ***cis-*Ru-1** analog with a *N*-heterocyclic carbene (NHC) ligand bearing photosensitive supersilyl ethers (***cis-*Ru-4**) was synthesized. This novel complex could be activated with 350 nm light and decomposed by UV-C irradiation. The catalyst provided excellent results for both options, the activation, and the self-destruction; however, a major drawback of this catalyst was the lengthy and difficult synthesis of the NHC ligand and its installation on the ruthenium complex. Recently, we discovered that latent metathesis catalysts-bearing phosphite ligands, for example, the commercially available catalyst ***cis-*Caz-1**, may be efficiently activated with UV-A and even visible light [56]. This discovery led to the design of novel phosphite containing latent metathesis catalysts. One of these complexes, ***cis*-Ru-5**, was designed to possess a chromatic orthogonal kill switch based on the more efficient photochemistry of the 2-nitrobenzyl group (compared to the less efficient supersilyl photochemistry) [57]. The destruction mechanism of the complex, although not completely elucidated, involves the cleavage of the photosensitive 2-nitrobenzyl groups (Figure 7).

**Figure 8:**
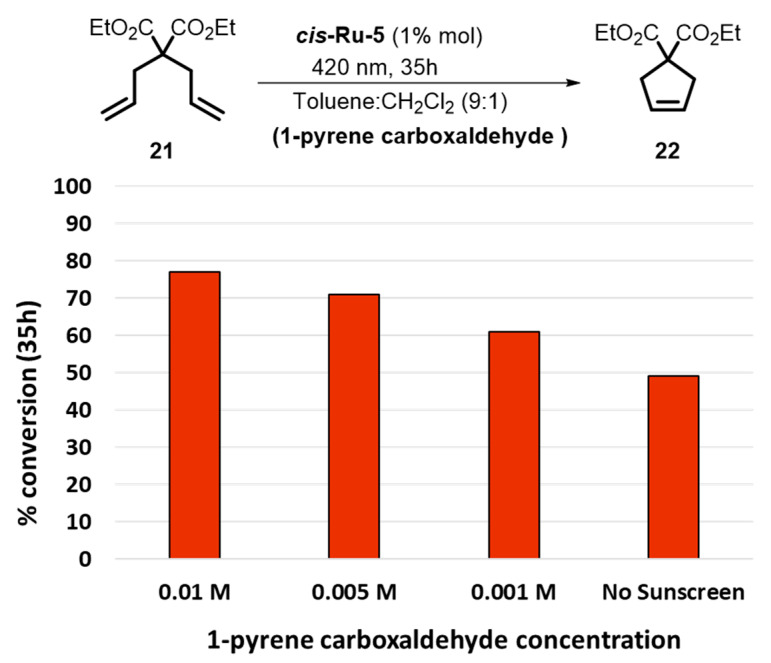
RCM of diethyl diallyl malonate using ***cis*-Ru-5** in the presence of an external solution of 1-pyrenecarboxaldehyde as a sunscreen in different concentrations.

Unlike the procedure required to make ***cis*-Ru-4**, the preparation of the 2-nitrobenzylphosphite ligand is simple and straightforward using commercially available reagents, and the installation of the phosphite ligand on the ruthenium metal center to generate ***cis-*Ru-5** is rapid and efficiently completed under mild conditions. Additionally, catalyst ***cis*-Ru-5** can be activated with visible light and destroyed by a wider range of UV light (although much faster with UV-C). While studying the catalytic activity of ***cis*-Ru-5** for olefin metathesis reactions, we observed that when reactions are exposed to 420 nm light, the catalyst shows significant decomposition, and after four hours of irradiation, no active catalyst was left in the reaction mixture. This was explained by the fact that although the light emitted by the lamps is centered at 420 nm, UV-A light is also emitted, and this degrades the complex over time due to the self-destruction mechanism. Thus, we hypothesized that a UV-A sunscreen could be used to protect this catalyst. To test this premise, model RCM reactions of diethyl diallylmalonate were carried out with 1 mol% of ***cis*-Ru-5** and irradiated with 420 nm light in the absence and presence of different concentrations of an external solution of 1-pyrenecarboxaldehyde as a light filter (Figure 8).

**Scheme 5:**
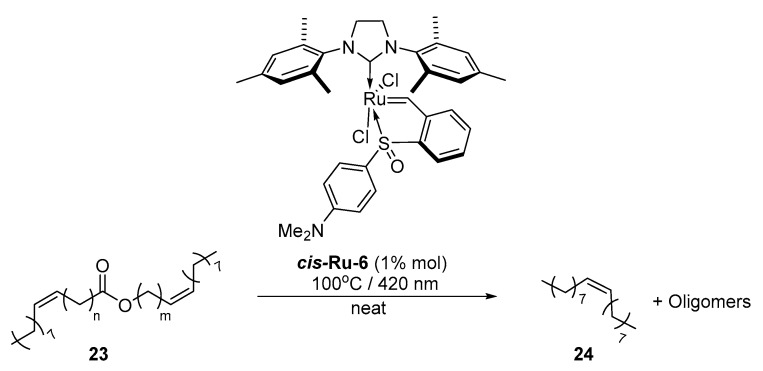
Self-metathesis of Jojoba oil catalyzed by photoswitchable sulfoxide catalyst ***cis*-Ru-6.**

Indeed, as shown in Figure 8, 1-pyrenecarboxaldehyde blocked the destructive UV-A light emitted by the lamps and significantly improved the reaction conversions. As a result of adding the external filter solution, conversion levels increased from 49% to 77%, and even after 35 h of irradiation, active catalyst was still observed in the reaction mixture, once again demonstrating the efficiency of 1-pyrenecarboxaldehyde as a photoprotective agent for UV-A light photochemical reactions. As demonstrated above, a judicious use of filter molecules can promote selectivity in photochemical reactions by blocking undesired reaction pathways. On the other hand, the same phenomenon may also impede the execution of the target reaction. Recently, we reported the self-metathesis of neat Jojoba oil using a sulfoxide-based photoswitchable metathesis catalyst ***cis*-Ru-6** developed in our laboratory (Scheme 5) [58].

When the complex was thermally activated by heating the Jojoba oil to 100 °C, the reaction proceeded smoothly, yielding the valuable C18 fragment and the ester oligomers (along with minor by-products caused by double-bond migration). However, when the same reaction mixture was irradiated with visible light (420 nm), no sign of metathesis could be observed. The raw Jojoba oil has a yellowish color that originates in natural impurities in the Jojoba seeds. Thus, we hypothesized that these impurities may disrupt the photoactivation of the metathesis catalyst by blocking the incident light by an undesired sunscreen effect. This issue was opportunely solved by filtration of the Jojoba oil through activated charcoal and removal of the colored impurities. Indeed, after filtration, the photochemical self-metathesis of Jojoba oil was carried out successfully. To further investigate this effect, a control experiment was conducted: 10% mol 1-nitropyrene (a dye that absorbs in the UV-A and visible light region) was added to a solution of diethyl diallyl malonate (**21**) and 1% mol ***cis-*Ru-6** in toluene. Indeed, the photoinduced metathesis was arrested in the presence of the dye, while the thermally activated reaction (80 °C) produced the desired RCM product.

## 3. Conclusions

To summarize, in this short review, a new method geared toward achieving selectivity in photochemical reactions using simple and available molecular filters was presented. The generality of the technique was demonstrated with two different sunscreen molecules: phenanthrene for reactions that are induced by UV-C light and 1-pyrenecarboxaldehyde for photochemistry with UV-A light. This methodology was applied in the selective PPG removal of the more absorbing 2-nitrobenzyl group over supersilyl ethers with UV-C light. Meanwhile, with UV-A light, 2-nitrobenzyl could be now protected with 1-pyrenecarboxaldehyde dye to allow a catalytic CM reaction in the all-photochemical synthesis of coumarin derivatives and also to increase the efficiency of a metathesis catalyst with a 2-nitrobenzyl based chromatic orthogonal self-destruction switch. Phenanthrene was also used to modulate a photochemically divergent reaction and enabled the selective photochemical synthesis of valuable butenolide and levulinate scaffolds from simple starting materials.
